# Supplementary material for: Rapid three-dimensional multiparametric MRI with quantitative transient-state imaging
Source: Sci Rep. 2020 Aug 13;10:13769. doi: 10.1038/s41598-020-70789-2 (PMC7427097; doi:10.1038/s41598-020-70789-2)
Supplement: Supplementary file 1 — Supplementary Information. [file 41598_2020_70789_MOESM1_ESM.pdf]

Rapid three-dimensional multiparametric MRI with quantitative transient-state imaging

Pedro A. Gómez [1], Matteo Cencini [2,3], Mohammad Golbabaee [5], Rolf F. Schulte [6],
Carolin Pirkl [1,6], Izabela Horvath [1,6], Giada Fallo [2,3], Luca Peretti [2,3],
Michela Tosetti [3,4], Bjoern H. Menze [1], Guido Buonincontri [3,4]

[1] Computer Science, Technical University of Munich, Munich, Germany [2] University of Pisa, Pisa, Italy
[3] Imago7 Foundation, Pisa, Italy [4] IRCCS Stella Maris, Pisa, Italy [5] University of Bath, Bath, UK [6] GE Healthcare, Munich, Germany

Supplementary material

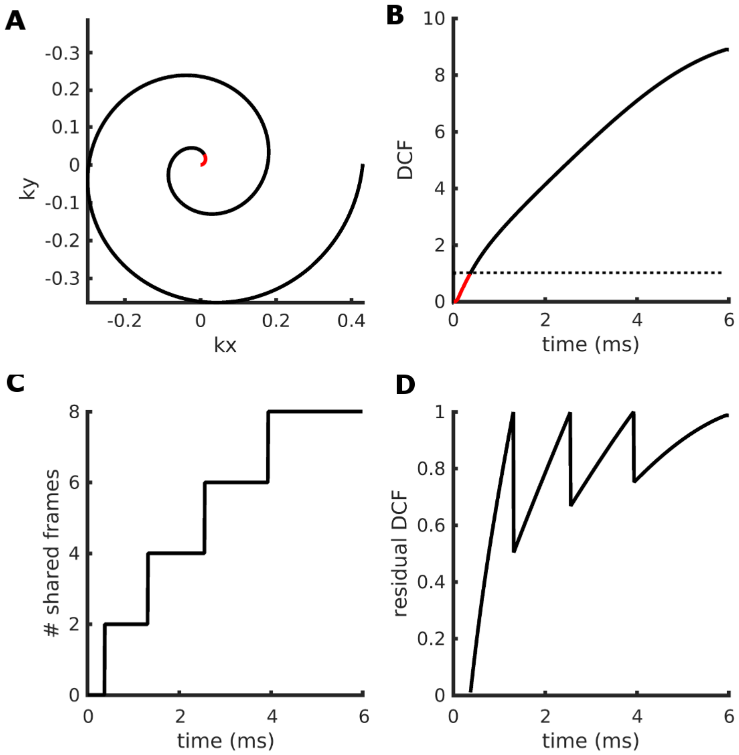

Supplementary Figure 1: Sketch of the k-space view sharing algorithm. *Sketch of the algorithm using an undersampled spiral trajectory. The oversampled part of k-space is in marked in red in panel A) with the sampling trajectory and in panel B) with its corresponding DCF. In the oversampled (red) area, the algorithm applies density compensation as in regular gridding. In the undersampled area (black), data is shared with n neighboring frames proportionally to the DCF (panel C). The residual DCF is then applied to the borrowed frames (panel D).*

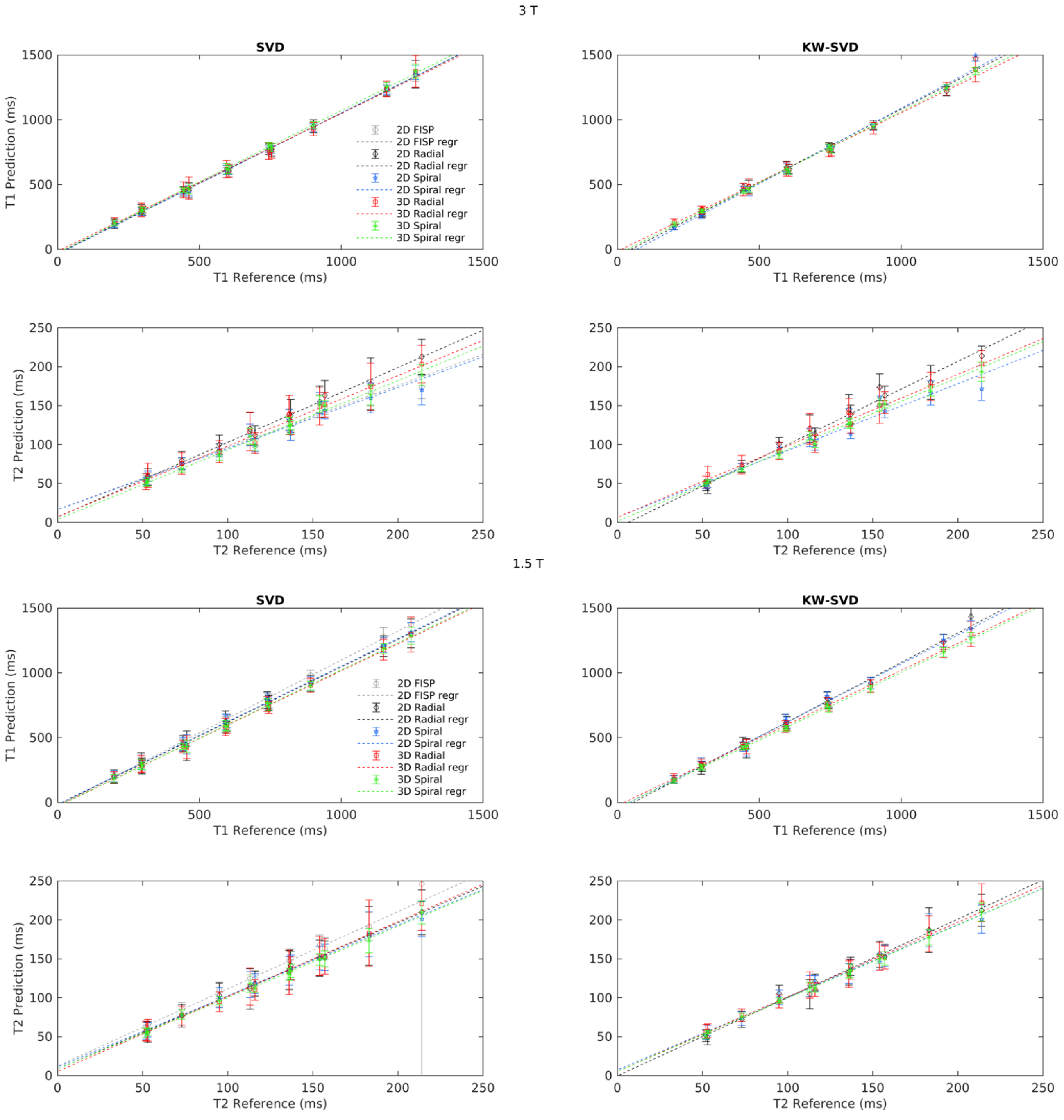

Supplementary Figure 2: Multipath neural network quantification results versus gold standard reference. *Quantification results at 1.5T and 3T for all readout trajectories with and without k-space weighted view-sharing.*

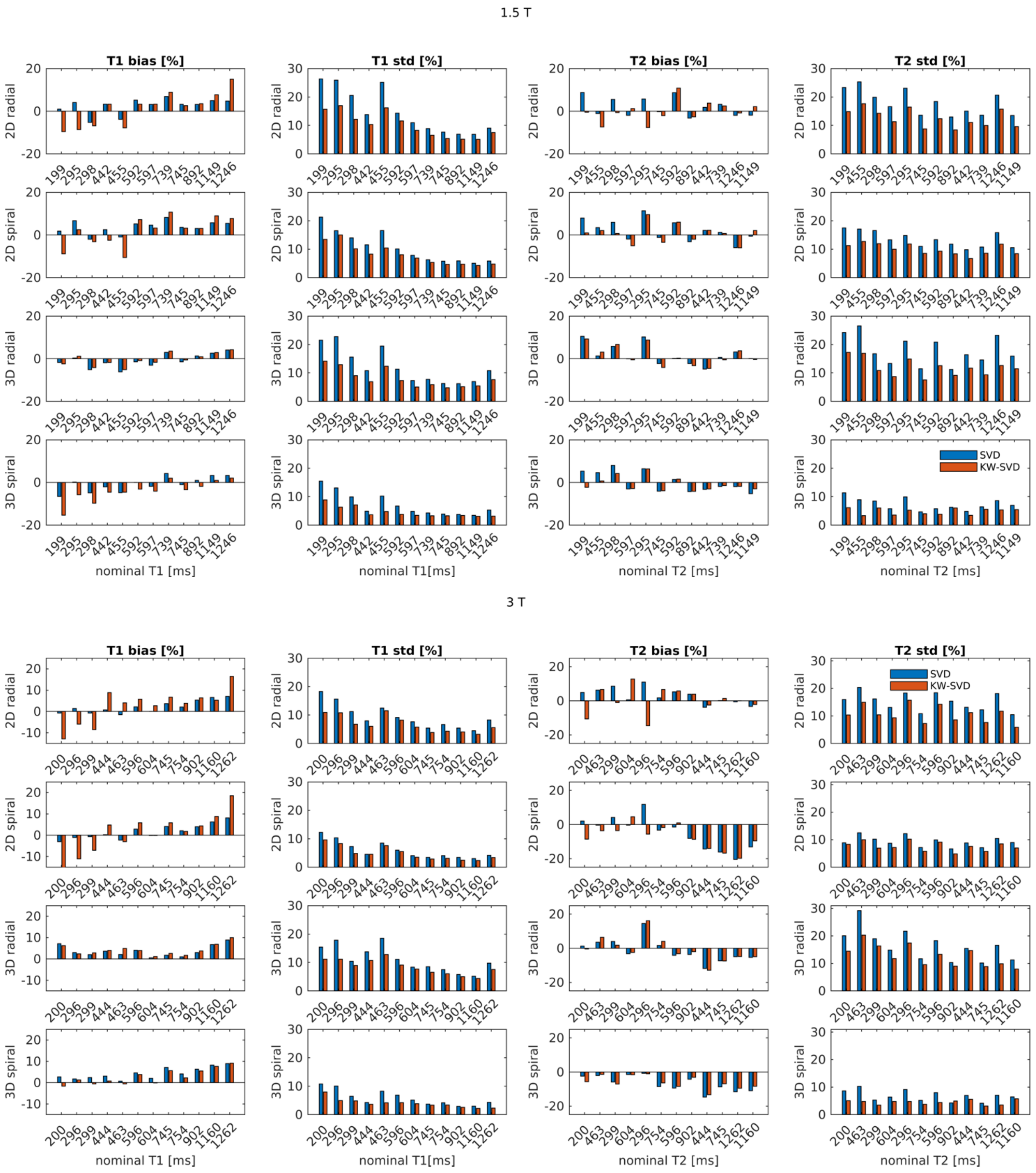

Supplementary Figure 3: Multipath neural network quantification bias and standard deviation.

Quantification bias and standard deviation at 1.5T and 3T for all readout trajectories with and without k-space weighted view-sharing.

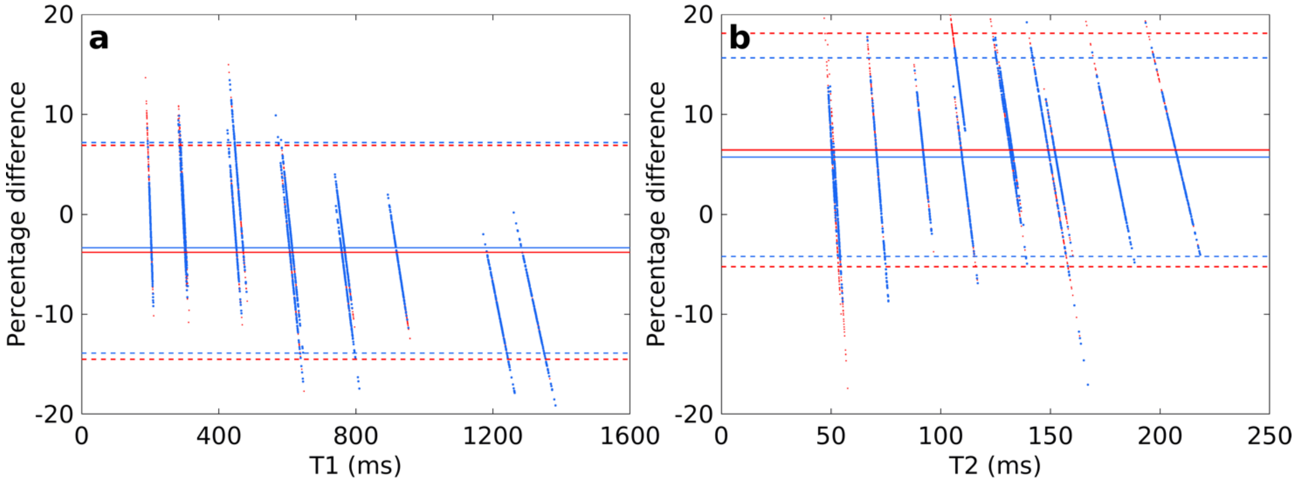

Supplementary Figure 4: Multipath neural network quantification validation for high-resolution mapping. *The plot compares percentage differences against the gold standard reference for the standard 3D spiral (red) and the high-resolution version (blue). Parameter inference in both cases was obtained using the NN multipath architecture.*

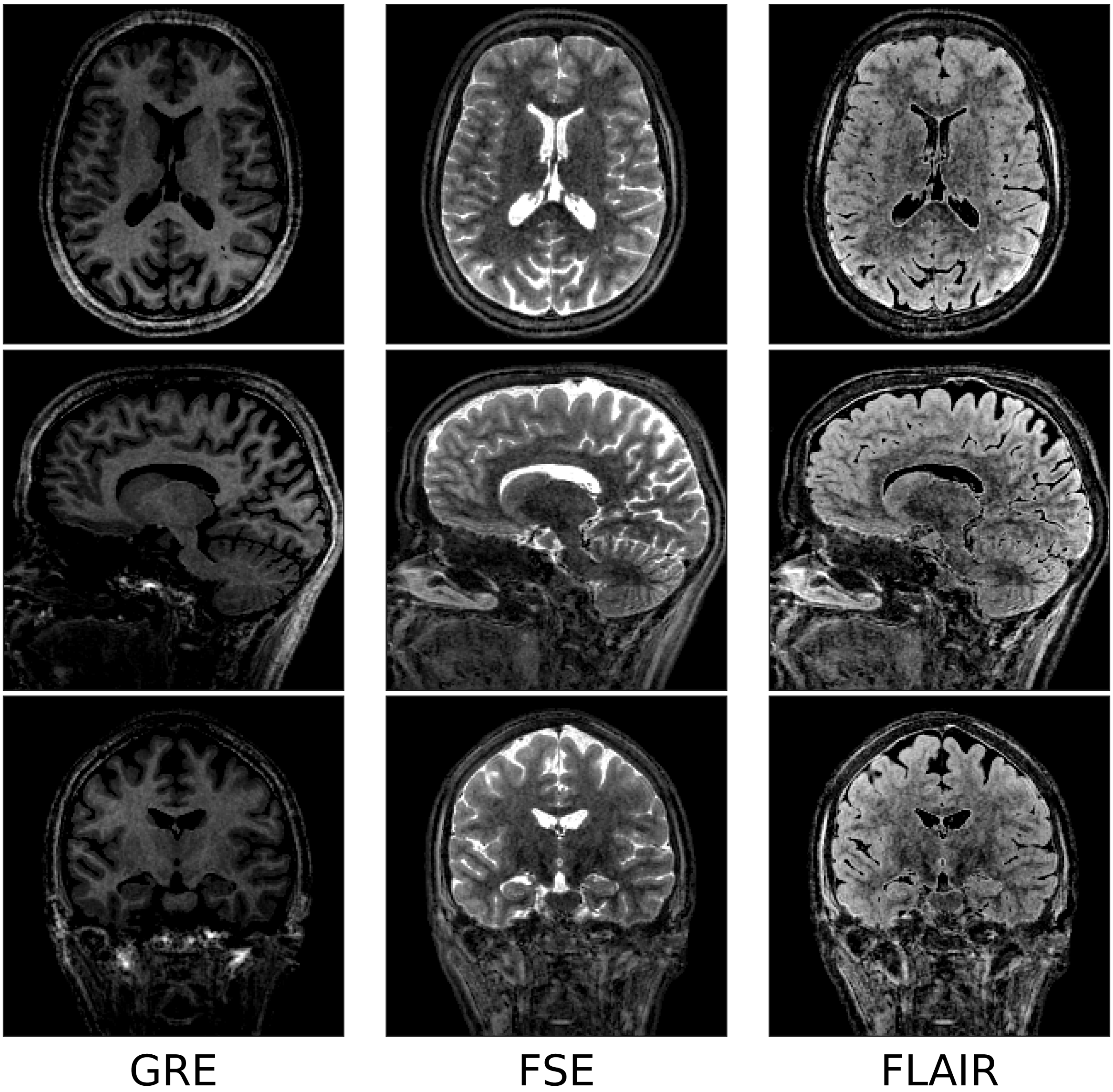

Supplementary Figure 5: Contrast-synthesis. *The inclusion of PD enables the creation of synthetic contrasts.*
